# Hologenome analysis of two marine sponges with different microbiomes

**DOI:** 10.1186/s12864-016-2501-0

**Published:** 2016-02-29

**Authors:** Taewoo Ryu, Loqmane Seridi, Lucas Moitinho-Silva, Matthew Oates, Yi Jin Liew, Charalampos Mavromatis, Xiaolei Wang, Annika Haywood, Feras F. Lafi, Marija Kupresanin, Rachid Sougrat, Majed A. Alzahrani, Emily Giles, Yanal Ghosheh, Celia Schunter, Sebastian Baumgarten, Michael L. Berumen, Xin Gao, Manuel Aranda, Sylvain Foret, Julian Gough, Christian R. Voolstra, Ute Hentschel, Timothy Ravasi

**Affiliations:** KAUST Environmental Epigenetic Program (KEEP), King Abdullah University of Science and Technology, Thuwal, 23955-6900 Kingdom of Saudi Arabia; Division of Biological and Environmental Sciences & Engineering, King Abdullah University of Science and Technology, Thuwal, 23955-6900 Kingdom of Saudi Arabia; School of Biotechnology and Biomolecular Sciences & Centre for Marine Bio-Innovation, University of New South Wales Sydney, Sydney, Australia; Department of Computer Science, University of Bristol, 24 Tyndall Ave, Bristol, UK; Red Sea Research Center, King Abdullah University of Science and Technology, Thuwal, 23955-6900 Kingdom of Saudi Arabia; Computer, Electrical and Mathematical Sciences and Engineering Division, King Abdullah University of Science and Technology, Thuwal, 23955-6900 Kingdom of Saudi Arabia; Computational Bioscience Research Center, King Abdullah University of Science and Technology, Thuwal, 23955-6900 Kingdom of Saudi Arabia; Center for Desert Agriculture, King Abdullah University of Science and Technology, Thuwal, 23955-6900 Kingdom of Saudi Arabia; Imaging and characterization Lab, King Abdullah University of Science and Technology, Thuwal, 23955-6900 Kingdom of Saudi Arabia; Division of Evolution, Ecology and Genetics, Research School of Biology, The Australian National University, Canberra, ACT 2601 Australia; GEOMAR Helmholtz Centre for Ocean Research, RD3 Marine Microbiology and Christian-Albrechts University of Kiel, Düsternbrooker Weg 20, D-24105 Kiel, Germany; Present address: APEC Climate Center, Busan, 48058 South Korea

**Keywords:** Sponge, *Stylissa carteri*, *Xestospongia testudinaria*, Innate immune system, Host, Microbial symbionts, Hologenome

## Abstract

**Background:**

Sponges (Porifera) harbor distinct microbial consortia within their mesohyl interior. We herein analysed the hologenomes of *Stylissa carteri* and *Xestospongia testudinaria*, which notably differ in their microbiome content.

**Results:**

Our analysis revealed that *S. carteri* has an expanded repertoire of immunological domains, specifically Scavenger Receptor Cysteine-Rich (SRCR)-like domains, compared to *X. testudinaria*. On the microbial side, metatranscriptome analyses revealed an overrepresentation of potential symbiosis-related domains in *X. testudinaria*.

**Conclusions:**

Our findings provide genomic insights into the molecular mechanisms underlying host-symbiont coevolution and may serve as a roadmap for future hologenome analyses.

**Electronic supplementary material:**

The online version of this article (doi:10.1186/s12864-016-2501-0) contains supplementary material, which is available to authorized users.

## Background

Microbial symbionts are being increasingly recognised as deeply integral components of multicellular organisms that affect core host functions such as development, immunity, nutrition, and reproduction [1]. The holobiont (synonym with “metaorganism”, and defined as the host organism and its collective microbial community) [2] is thus considered a biological unit of natural selection (the “hologenome theory”) [3]. However, the molecular mechanisms (e.g., immune system evasion and tolerance) that have resulted in these symbiotic partnerships are poorly understood.

Sponges (Porifera) represent one of the oldest, still extant animal phyla. Fossil evidence dating back 580 million years ago shows their existence in the Precambrian long before the radiation of all other animal phyla [4]. Sponges are globally distributed in all aquatic habitats from warm tropical reefs to the cold deep sea and are even present in freshwater lakes and streams. As sessile filter feeders, they pump many thousands liters of water per day through the aquiferous canal system that is embedded within the sponge body and are constantly exposed to a plethora of microorganisms from the environment [5]. Many species are colonised by dense and diverse microbial consortia that are contained extracellularly within the mesohyl matrix (“high microbial abundance” (HMA)), while other species are nearly devoid of microorganisms (“low microbial abundance” (LMA)) [6–8].

**Fig. 1:**
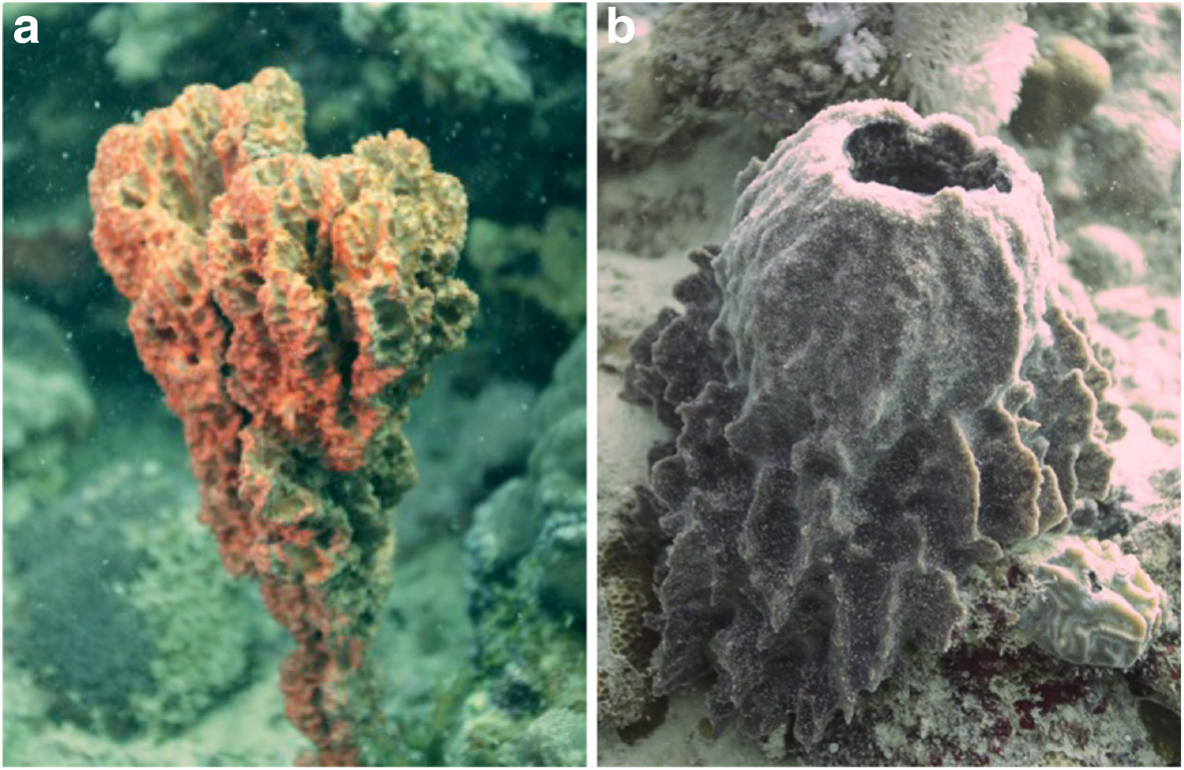
Sponge species. Underwater images of *Stylissa carteri* (**a**) and *Xestospongia testudinaria* (**b**) taken by Michael L. Berumen

To investigate factors involved in sponge-microbe interactions, we herein sequenced and analysed hologenome data including genome, transcriptome, and metatranscriptome of *S. carteri* (an LMA sponge and hereafter referred to as “*SC”*) and *X. testudinaria* (an HMA sponge and hereafter referred to as “*XT”*) (Fig. 1, Additional file 1 and Additional file 2). These two sponges were collected from the same habitat, which ensured systematic comparison by minimizing environmental effect such as different planktons and temperature. *SC* is the first species in the order Halichondrida to have its genome sequenced while *XT* (order Haplosclerida) is the first HMA sponge to have its genome sequenced [8]. Thus, our work provides a unique and valuable resource for future studies.

## Results and discussion

### Genome assembly and gene annotation

Our assemblies of the *SC* and *XT* genomes yielded 97,497 and 97,640 scaffolds with base-level coverages of 109 X and 59 X, respectively (Additional file 3). The estimated genome sizes obtained from our assemblies were comparable to experimentally determined genome sizes measured by flow cytometry (386.31 and 161.37 Mbp for *SC* and *XT*, respectively, see Methods). 26,967 and 22,337 gene models of high quality were predicted covering 94.5 and 94.3 % of the core eukaryotic genes for *SC* and *XT*, respectively (see Methods). Comparison to publicly available sponge gene models from draft genomes or transcriptomes [9–11] shows that our gene models have reasonable quality in terms of the number of coding sequences (CDSs), the representation of core eukaryotic gene set [12], the number of genes with protein domains, and the number of protein domains (Additional file 4).

### Expansion of innate immunological domains in sponge hosts

**Fig. 2:**
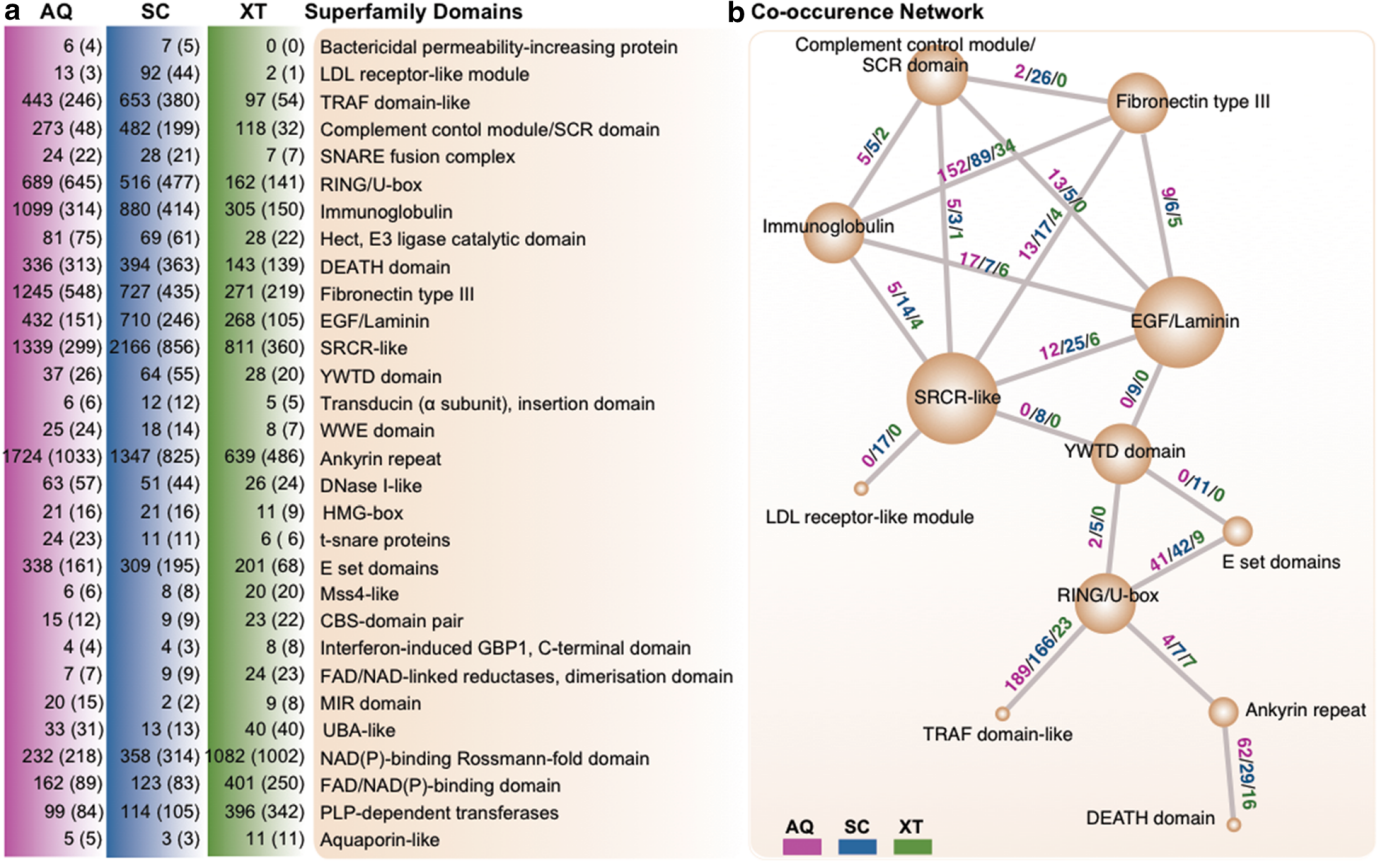
Domain expansion and contraction in LMA vs. HMA sponges. Innate immune- and symbiosis-related protein domains were selected from the functional annotation. **a** The number of times a domain occurs in each genome. Shown are domains found ≥ 1.5 times more in *SC* compared to *XT*, or vice versa. *AQ* is included for comparison. The domain occurrence is highly correlated in the LMA sponges (*AQ* and *SC*; Pearson correlation coefficient = 0.92). The numbers of genes containing the domains are shown in the bracket. **b** The co-occurrence of innate immune domains in sponges. Nodes represent domains, while the numbers above the edges indicate the number of proteins in which the two domains co-occur for each sponge (*the color key is shown at the bottom-left*). Each node size is proportional to the number of outgoing edges. Edges are shown only when the domain pairs were observed ≥ 5 times in ≥ 1 species

We checked for protein domains that were unusually over- or under-represented in the studied sponges compared to other eukaryotes compiled in the SUPERFAMILY database [13]. Most protein domains were neither over- nor under-represented, indicating that our gene models are comparable to those of other eukaryotes (Additional file 5). Both sponges (particularly *SC*) showed substantial expansions of immunological and receptor domains (Additional file 5) [14]. We focused our analysis on protein domains relevant to host-microbe interactions by using four keywords (“symbio,” “innate immunity,” “antimicrobial peptides,” and “antibacterial”) in functional annotations of the SUPERFAMILY database [13] (Fig. 2a and Additional file 6). We also included *Amphimedon queenslandica* (hereafter referred to as “*AQ*”) in this analysis because its genome has been stably annotated [9] and also because its status with respect to microbial load is well known (LMA, Sandie Degnan, personal communication). Furthermore, overall gene contents of *AQ* are comparable to our studied sponges: among 30,060 gene models for *AQ*, 17,567 genes contained 32,326 SUPERFAMILY domains (28,027 and 29,156 SUPERFAMILY domains from 17,074 and 17,664 genes for *SC* and *XT*, respectively. Additional file 4). Other sponges with public transcript models were excluded due to incompleteness of gene models as shown in Additional file 4 and lack of exact status of LMA and HMA.

Our results revealed that there had been a striking expansion of the Scavenger Receptor Cysteine-Rich (SRCR)-like domain in the three tested sponges compared to all other eukaryotes compiled in the SUPERFAMILY database (see Additional file 6 for selected taxa). One known function of SRCR-like domains is recognition of large and diverse patterns of macromolecules (e.g., modified low-density lipoprotein; LDL) on microbial surfaces and enhancement of the phagocytic clearance of microbes [7]. In mammals, malfunctions in SRCR-like-domain-containing proteins have been linked to diseases and bacterial/viral infections [15]. It has been suggested that a protein containing this domain in the Mediterranean sponge (*Petrosia ficiformis*) may function in the recognition of photosymbionts [16]. We found that the *SC* genome contains 2166 SRCR-like domains, which is the highest number found among the 427 eukaryotes compiled in the SUPERFAMILY database (average, 28 copies). Interestingly, the next-highest known copy number for this domain family is found in *AQ*, followed closely by the sea urchin, *Strongylocentrotus purpuratus* (1339 and 1328 copies, respectively). In contrast, *XT* was found to have 811 copies.

**Fig. 3:**
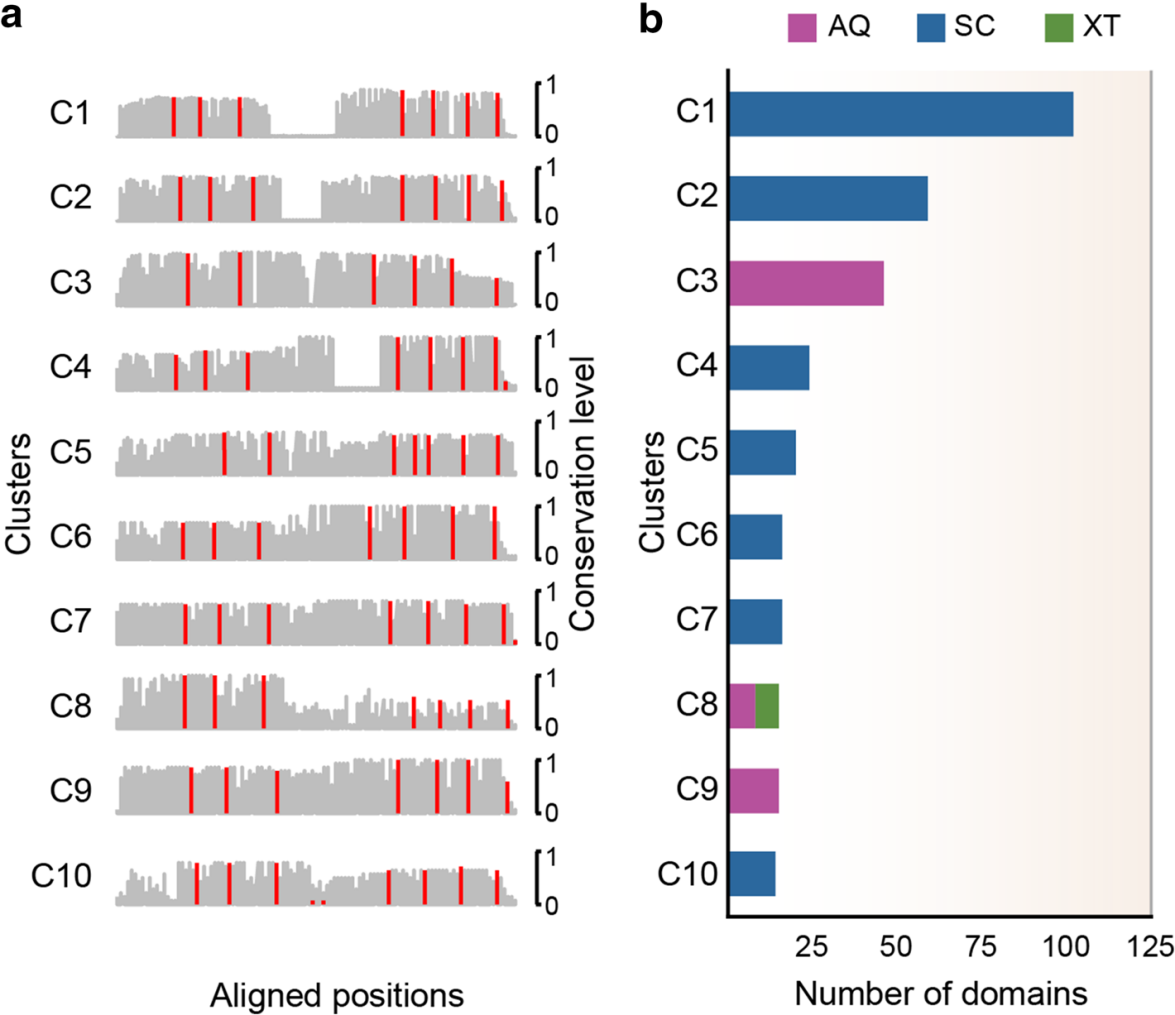
Diversity of sponge SRCR-like domains. **a** Clustering and sequence alignments revealed diverse primary structures of the sponge SRCR-like domains. The conservation levels of cysteine residues are shown in red, while those of other amino acids are shown in gray. Only the top ten largest clusters are shown. **b** Lineage-specific expansions of each cluster in (**a**). This analysis suggests that the SRCR-like domains had expanded in LMA sponges, possibly enabling the recognition of various microbial ligands

The SRCR-like domains also show unique combinations with other immune system domains in sponges (Fig. 2b). For example, SRCR-like domains co-occur with LDL receptor-related domains (i.e., the LDL receptor-like module and the YWTD domain [17]) only in *SC*. SRCR-like domains are also associated with a broad collection of immunoglobulin-like beta-sandwich-folds in the three sponges, but most prominently in *SC*; these associated domains include the fibronectin type III and immunoglobulin domains, which are involved in cell surface recognition [18]. Compared to *XT*, *AQ* and more notably *SC* have undergone considerable expansions in combinations of the SRCR-like and abovementioned domains. In the SRCR-like domains, most of the amino acid residues are highly variable except for certain key residues including specific cysteine residues that enable the SRCR-like domains to recognise a vast array of ligands [15]. Clustering of the SRCR-like domain sequences from the three sponges yielded a large number of groups (169 clusters with ≥ 5 domains) whose members showed distinct patterns in their cysteine residues and levels of sequence conservation (Fig. 3a; see Methods). Thus, these domains are characterised by great diversity at the sequence level. The clusters were also distinct from one another in terms of their species compositions and expansion levels (Fig. 3b). The largest clusters contained the SRCR-like domains of *SC* and *AQ*, indicating that these domains are diversified to a greater extent in these species than in *XT*.

Additionally, we observed the expansions of other innate immune domains in *SC* and *AQ* (Fig. 2a). Among the selected examples are bactericidal permeability-increasing proteins. These host-defending antibiotic molecules, which selectively kill gram-negative bacteria [19, 20], were found only in *AQ* and *SC*. High-mobility group (HMG)-box domains were also found to be expanded in *SC* and *AQ* over *XT*. HMG proteins are primarily nucleosome-binding proteins, but some members are released extracellular milieu and propagate danger signal upon infection and tissue damage to active innate and adaptive immune responses in higher eukaryotes [21–23], which is known as “alarmin” functions which sense exogenous microbe- or pathogen-associated molecular patterns (MAMPs/PAMPs) or endogenous danger-associated molecular patterns (DAMPs) and then modulate downstream immune responses. Although not listed in Fig. 3 due to our SUPERFAMILY-based annotation scheme [13], another set of alarmins, the NACHT domains (PF05729), were also found to be enriched in *AQ* (230 copies) and *SC* (64 copies) compared to *XT* (21 copies). This domain is a component of the nucleotide-binding domain and leucine-rich repeat (NLR) proteins, which are major intracellular pattern recognition receptors (PRRs) [14]. DEATH domains, which are often found in MYD88 and NLR proteins, and the TRAF domain-like domains, which functions downstream of the classic Toll/Toll-like receptor pathway, were also found to be enriched in *SC* and *AQ* [11, 14, 24] (Fig. 2). Notably, however, the copy number of the Toll/interleukin receptor domain, which is a component of another set of PRR proteins [25], did not follow the above-described pattern, with 8, 17, and 16 copies found in *AQ*, *SC*, and *XT*, respectively (Additional file 6).

Interestingly, consistent with its symbiont-containing status (Additional file 1), *XT* was enriched over *SC* and *AQ* in protein domains that contribute to controlling symbiosis in some eukaryotes (Fig. 2). These include GBP1, which has been associated with the parasitophorous vacuole (responsible for host defense) [26], and aquaporin, which controls pH and the salt concentration in the symbiosome compartment in legumes, corals, and sponges (a symbiotic interface between host and microbes) [27–30].

We observed further strong correlations in the patterns of protein domain expansion between the LMA sponges, *AQ* and *SC*. In contrast, fewer similarities were found between the protein domains of *AQ* and *XT*, even though these two species belong to the same order (Haplosclerida). Analyses of antimicrobial peptides on the sponge genomes (Additional file 7, Additional file 8, Additional file 9, Additional file 10, and Additional file 11) and evolutionary rates of protein domains (Additional file 7 and Additional file 12) also provided consistent results.

### Host-interaction factors in microbial symbionts

**Fig. 4:**
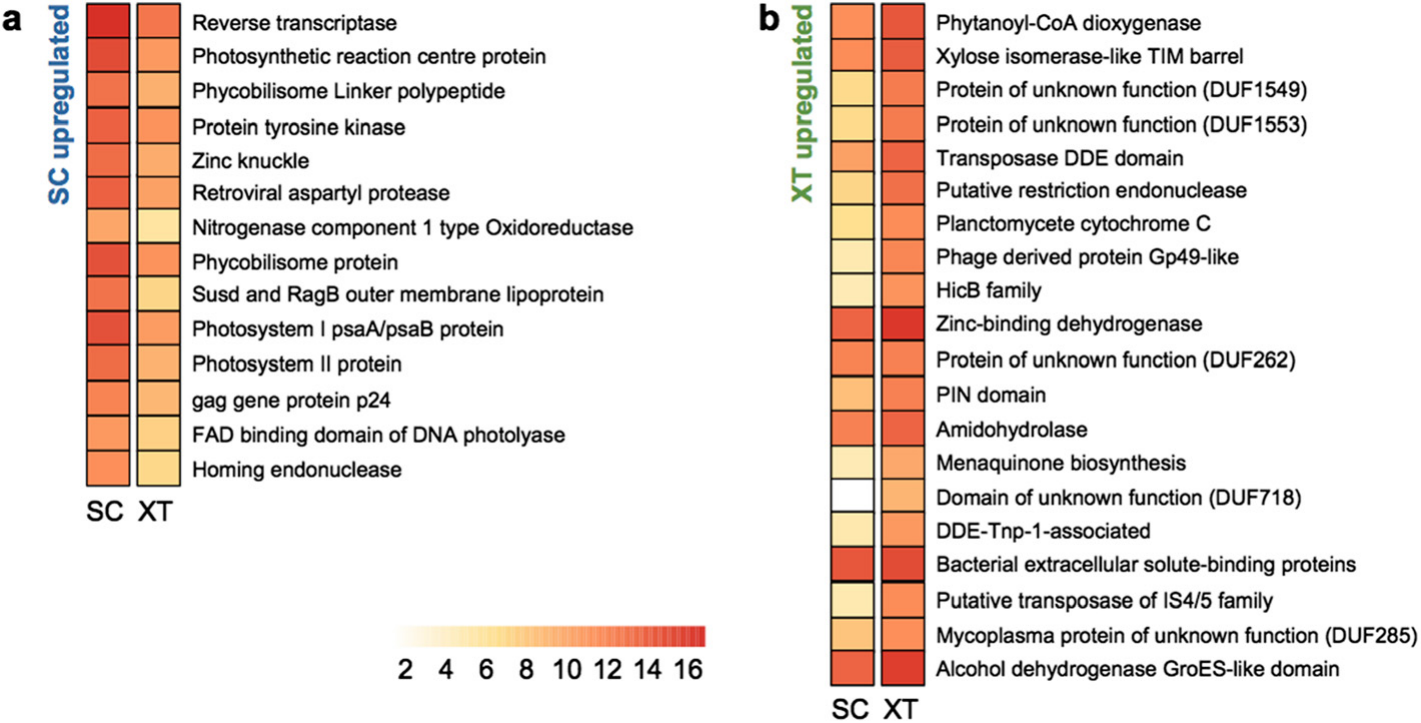
Comparison of the enriched protein domains in the sponge symbionts. Significantly enriched PFAM domains among the genes found to be differentially expressed in the studied sponges (false discovery rate < 0.05) are shown for (**a**) *SC* and (**b**) *XT*, along with the expression levels of the domain-containing genes. Colors in the heatmap represent log_2_-normalised expression levels

Previous studies showed that *SC* and *XT* harbor distinct microbial symbionts encompassing about 27 bacterial and archaeal phyla [31–35], but it is unclear how the unique microbiomes of LMA and HMA sponges are shaped in the context of the holobiont. We therefore analysed the metatranscriptomes of the microbial consortia in *SC* and *XT*. Since the metatranscriptome of *AQ* is not available, it could not be included in the present study. Although most well-known protein domains for symbiosis or pathogenesis [36–38] were not over-represented in any of the sponge symbionts, the fibronectin type III domain was among the most abundant domains in both sponge microbiomes, suggesting that this eukaryotic-like domain [37] may be a major contributor for the maintenance of host-microbe interactions (Additional file 13). Differential expression analysis of the microbiome genes identified several intriguing protein domains that were significantly over-represented in *XT* over *SC* (Fig. 4 and Additional file 14), including: the “Xylose isomerase-like TIM barrel” domain (PF01261), which is thought to be involved in the symbiosis of microbes with leguminous plants and the termite hindgut [39, 40]; the “HicB family” domain (PF05534), which is related to pilus formation and required for niche invasion [41]; the “PIN domain” (PF01850), which is found in the toxin-antitoxin operons of prokaryotes [42]; and the “Mycoplasma protein of unknown function” domain (PF03382), which has been detected in many pathogenic bacteria [43].

The sponge microbiomes also showed distinct community functions (Fig. 4 and Additional file 14). Consistent with our previous findings [33], light-harvesting functions were significantly enriched in the *SC* metatranscriptome, implying that photosynthesis is a major source of nutrients for the symbionts of *SC*. Additionally, virus-related functions were significantly enriched in the *SC* metatranscriptome, corroborating the idea that defence mechanisms against viruses, which are abundant in seawater, may be relevant to this community [37, 44]. On the other hand, the *XT* metatranscriptome was enriched for transposases, which may ensure the exchange of mobile genetic elements and help distribute selectable traits across diverse species [45].

## Conclusions

Sponges serve as important organisms for the study of host-microbe interactions in lower marine invertebrates. Our present work identified expansion of potential immune system components especially SRCR-like domains in marine sponges compared to other eukaryotes probably as a result of coevolution with residing microbes. We also identified that sponge genomes expanded protein domains to a different extent by the microbiome contents. Our findings on the putative molecular underpinnings of sponge-microbe interactions provide a foundation for a better understanding of the mechanisms of host-microbe interactions in early branching metazoans [7, 46].

## Methods

### Ethics statement

This study did not include protected or endangered species and require ethical approval.

### Sample collection

Specimens of *SC* and *XT* were collected from 2010 to 2013 via SCUBA at Fsar Reef (22.228408 N, 39.028187E) on the Red Sea coast of Saudi Arabia (Additional file 2). Sponge samples were collected at a depth of 13-14 m. Immediately (i.e., on board the vessel), a scalpel was used to cut the sponges into 2 to 3 cm^3^ pieces, and the pieces were washed three times with autoclaved artificial seawater (ASW). Thereafter, the samples were either frozen in dry ice for DNA extraction, or incubated overnight at 4 °C in RNAlater (Ambion, USA) and stored at − 80 °C for RNA extraction. These cooled samples were transported to the laboratory for experiments.

### Transmission electron microscopy

The 3 mm^3^ pieces of sponge were fixed with 2.5 % glutaraldehyde in seawater for ≥ 48 h, treated with reduced osmium (1:1 mixture of 2 % aqueous potassium ferrocyanide) for 1 h as described previously [47], gradually dehydrated using an ethanol series (70, 80, 90, 95, and 100 %), and embedded in Epoxy resin. Thereafter, 80 to 120 nm-thick sections were collected on copper grids and contrasted with lead citrate. Imaging was performed using a Tecnai transmission electron microscope operating at 120 kV (FEI, USA). Images were recorded on a 2 K × 4 K CCD camera (Gatan Inc., USA).

### DNA extraction

Sponge tissues were ground under liquid nitrogen, and genomic DNA was extracted from 20 to 30 mg ground tissue using an All-PrepDNA kit (Qiagen, Germany). The extracted DNA was eluted with 100 μl of water, and its quality and quantity were measured using a NanoDrop 8000 spectrophotometer (Thermo Scientific, USA). To test the level of bacterial DNA in the extracted DNA, PCR was performed using the Qiagen PCR Master Mix solution (Qiagen, Germany) and two primer pairs (Bac27F, 5’-AGAGTTTGATCMTGGCTCAG-3’ and Bac1492R, CGGTTACCTTGTTACGACTT; and COX1-D2, AATACTGCTTTTTTTGATCCT GCCGG and COX1-R1 TGTTGRGGGAAAAARGTTAAATT). The cycling conditions consisted of 15 min at 95 °C, followed by 30 cycles of 95 °C for 30 s, 56 °C for 90 s and 72 °C for 90 s, and a final extension of 10 min at 72 °C. The samples were resolved by 1 % agarose gel electrophoresis. The DNA integrity was checked, and samples that showed a brighter band for bacterial DNA compared to sponge genomic DNA were excluded from further analysis.

We additionally performed whole-genome amplification of isolated sponge cells from *SC* and *XT* (Additional file 2). The sponges were cut into 0.5 cm^3^ pieces, rinsed three times with cold calcium-magnesium-free (CMF)-ASW (0.55 M NaCl, 12 mM KCl, 6.3 mM Na_2_S0_4_, 5 mM Tris–HCl, 5 mM EDTA) at a 1:10 ratio of sponge:CMF-ASW, and agitated at 100 rpm overnight in fresh CMF-ASW. All liquid and the remaining sponge pieces were passed through a 70 μm Nitex filter (Fisher Scientific, UK), and each sample was centrifuged (700 g for 5 min at 4 °C). The pellet was washed with 10 ml of ASW, centrifuged, and suspended in 2 ml ASW. One ml of sample was gently layered atop a 30:50:70 % Percoll gradient in a 15 ml Falcon tube (VWR International, USA), and the sample-loaded gradient was centrifuged at 400 x g for 15 min at 4 °C. Each gradient layer was individually pipetted to a separate 2 ml tube and subjected to microscopic analysis. The layers representing 30:50 and 50:70 % Percoll were found to contain the most sponge cells and the fewest bacterial cells. These layers were washed with 5 ml ASW (300 x *g* for 5 min) and suspended in 200 μl 1xPBS. Micromanipulators (Narishige, Japan) were used to collect 15–30 sponge cells, which were dispensed to 3 μl sterile 1x PBS and subjected to whole-genome DNA amplification using an REPLI-g Midi kit (Qiagen, Germany). Briefly, 3.5 μl of Buffer D2 (83 mM DTT, 917 mM Reconstituted Buffer DLB) and 3 μl of cells in 1 x PBS were vortexed, briefly centrifuged, and incubated for 10 min on ice. Stop solution (3.5 μl) was added, and the sample was vortexed and then briefly centrifuged to yield denatured DNA. A master mix was made by combining 1 x SYBR Green, nuclease free H_2_0, REPLI-g Midi Reaction Buffer and REPLI-g Midi DNA polymerase (as per the instructions), and 50 μl of this master mix was added to 10 μl of the denatured DNA. The samples were incubated on a Real-Time PCR 7900 (Applied Biosystems, USA) at 30 °C for 16 h followed by 3 min at 65 °C (to inactivate the polymerase). Each sample was then analysed for bacterial contamination (as described above) and then stored at − 20 °C until use.

### Extraction of mRNA

Total RNA was extracted as described by Moitinho-Silva et al. [33], and sponge mRNA was isolated from the total RNA (100 μg) using a Poly(A) Purist MAG kit (Ambion, USA) with two rounds of poly(A) purification. The isolated sponge mRNA was linearly amplified using a MessageAmp II-Bacteria kit (Ambion, USA) as described, except that we omitted the polyadenylation of the template RNA (which is required only for prokaryotic RNA). RNA integrity was analysed using an Experion System (Bio-Rad, USA), and the isolated sponge mRNA was stored at − 80 °C until use.

### Microbiome RNA extraction

The metatranscriptome of each sponge was extracted as described by Moitinho-Silva et al. [33].

### Estimation of sponge genome size

Fresh sponge tissues were rinsed three times in filtered (0.22-μm, 142-mm Express Plus filters; Millipore, USA) seawater, fixed in 95 % ethanol and stored at − 20 °C. Small pieces of ethanol-preserved sponge (0.5–1 cm^3^) were subjected to two different nuclear suspension approaches, both involving the standard protocol of the CyStain® PI absolute T kit (Partec GmbH, Germany). For the first (tissue-grinder-based) approach, a piece of sponge was placed in a cryotube, incubated for 15 min in extraction buffer, and mashed with a tissue grinder for 1 min. The sample was filtered through a 40 μm nylon mesh filter, 250 μl of sample was combined with 1.25 ml (5 volumes) of staining solution (staining buffer + propidium iodide + RNase), and the mixture was incubated in the dark for 60 min. As a control, chicken erythrocytes (*Gallus gallus domesticus*, 2C = 2.45 pg) were included in the same tube and analysed in parallel with the sponge sample. For the second (bead-beating-based) approach, the sample was placed in a cryotube, incubated in extraction buffer (Partec GmbH, Germany) for 15 min, and homogenised with an MP FastPrep 24 machine (MP Biomedicals, USA) for 10–20 s (4.0 M/s, 2 ceramic beads). All samples were analysed using a BD Canto II flow cytometer (BD Biosciences, USA) with a 488 nm laser (to excite the PI) and a 585/42 band-pass emission filter.

Both methods yielded very similar genome sizes. For *SC*, the first and second protocols yielded haploid genome sizes of 0.395 pg (386.31 Mbp) and 0.39 pg (381.42 Mbp), respectively. For *XT*, both protocols yielded haploid genome sizes of 0.165 pg (161.37 Mbp).

### High-throughput sequencing

Genomic DNA and RNA libraries were prepared using the TruSeq kit (Illumina, USA). For mate-pair library preparation, a Nextera kit (Illumina, USA) was used for fragmentation, size selection, and circularisation, and then a TruSeq kit was used for end repair and adapter ligation. HiSeq2000 technology (Illumina, USA) was used for paired-end and mate-pair sequencing; 454 and Ion proton sequencing were conducted using standard protocols (Additional file 2). All sequencing was performed in the KAUST Bioscience Core Lab (Saudi Arabia).

### *De novo* assembly of sponge genomes and transcriptomes

The low-quality ends of short Illumina reads (spanning from the first base with Q-score < 20 up to the 3’ end) and sequencing adapters were trimmed. Long reads obtained from 454, Ion PGM, and Ion proton sequencing were split at each low-quality base (Q-score < 20), such that all bases in each split sequence had Q-scores ≥ 20.

The preprocessed genomic reads obtained using the different platforms were assembled with Velvet v1.2.09 [48], using *k*-mers from 55 to 75 with steps of 10. The Velvet assembly with *k* = 65 was selected, as it produced the longest scaffold N50. Transcriptomes were assembled with ABySS v.1.3.4 [49] and Trans-ABySS v.1.4.4 [50], using *k*-mers from 45 to 75 with steps of 10; these programs were chosen because benchmark tests [51, 52] showed that it yielded a higher accuracy than other *de novo* assemblers. Genomic scaffolds were further assembled using the transcriptomes, by the L_RNA_SCAFFOLDER [53]. After discarding short scaffolds (<800 bp) based on the genome annotation guideline [54], our analysis yielded 97,497 and 97,640 scaffolds for *SC* and *XT*, respectively. The statistics for our genomic and transcriptomic assemblies are summarised in Additional file 3. To obtain the base-level and mean coverages for each scaffold, we aligned the reads to the relevant scaffolds, and analysed them using BWA [55], SAMtools [56], BEDTools [57], and custom Java scripts. The mean base-level coverages of the *SC* and *XT* genomes were 109 X and 59 X, respectively. The host genome sizes for *SC* and *XT*, which were roughly estimated using scaffolds with GC % < 50, were 407.44 and 173.78 Mbp, respectively.

### Gene annotation

MAKER2 was used to annotate the gene models [58]. Assembled transcriptome contigs were used as the mRNA evidence, while proteins from the *Amphimedon queenslandica* (*AQ*), CEGMA, and UniProtKB/Swiss-Prot databases were used as protein homology evidence [12, 59]. Augustus (trained with the gene model from *AQ*) and SNAP were used as *ab initio* gene predictors inside the MAKER2 pipeline [60, 61]. Gene models with an Annotation Edit Distance (AED) score ≤ 0.75 from MAKER2 were selected.

To increase the authenticity of each predicted gene model, we tagged them as eukaryotic (E), prokaryotic (P), or unknown (X). A gene was tagged as “E” if the protein product had a hit to any eukaryotic gene (*e*-value < 10^−4^) in the NCBI non-redundant (nr) database, as assessed using Blastp [62]. A gene was tagged as “P” if it had a significant hit (*e*-value < 10^−4^) to prokaryotic genes without any eukaryotic gene hit. A gene was tagged as “X” if it lacked any significant hit. The statistics and properties of the genes identified with each tag are summarized in Additional file 3 and Additional file 15, respectively. We used only “E” genes for our downstream analysis (26,967 and 22,337 genes for *SC* and *XT*, respectively), because they were considered to represent *bona fide* host genes.

The completeness of each assembly was measured using CEGMA v2.4 [12], which revealed that 73 and 81 % of 458 Core Eukaryotic Genes (CEGs) were completely or partially present in the genomes of *SC* and *XT*, respectively. However, as reported in Smith et al. [63], these numbers can differ depending on the utilised search algorithm. Accordingly, we also used Blastp to search 458 CEGs against the sponge gene models, setting the *e*-value threshold to 10^−4^. This analysis indicated that 433 (94.5 %) and 432 (94.3 %) CEGs had homologs in *SC* and *XT*, respectively.

The quality of predicted gene models was assessed by comparing to those of publicly available Porifera dataset (Additional file 4). Gene models from the draft genomes were used for *AQ* [9], *SC*, and *XT*. Transcriptome assemblies of eight sponges (*Aphrocallistes vastus*, *Chondrilla nucula*, *Corticium candelabrum*, *Ircinia fasciculata*, *Petrosia ficiformis*, *Pseudospongosorites suberitoides*, *Spongilla lacustris*, and *Sycon coactum*) were retrieved from Riesgo et al. [11]. Transcript models for other sponges (*Ephydatia muelleri*, *Leucosolenia complicata*, *Oscarella carmela*, *Oscarella sp*, *Sycon ciliatum*) were retrieved from Compagen [10]. The CDSs of sponges except for *AQ*, *SC*, and *XT* were obtained by applying TransDecoder [64] and cd-hit-est [65] with default setting. Blastp [62] were performed for sponge CDSs against 458 CEGMA core gene set [12] with the threshold 10^−4^. SUPERFAMILY domains were annotated using Interproscan v5. RC7 [66].

### Functional annotation of genes

We annotated SUPERFAMILY and PFAM domains using InterProScan v5. RC7 [66]. The gene ontology (GO) terms were assigned to proteins harboring SUPERFAMILY and PFAM domains using dcGO [67] and InterProScan, respectively. Blast searches of the predicted sponge proteins were performed against the NCBI nr database, and homologs were identified with an *e*-value threshold of 10^−4^. The GO terms of the identified homologs were retrieved from the NCBI database and transferred to sponge genes using a custom Python script. Whole-genome over/under-representations of GO terms were ranked using Z-scores calculated from a background distribution generated for each annotated GO term (composed of dcGO results from 382 species found in the SUPERFAMILY library as of June 1, 2014). Due to redundancy among the SUPERFAMILY and PFAM domains and the more comprehensive functional annotation of the former by dcGO, we used the SUPERFAMILY domains for our analysis of the sponge domain repertoire.

### Analysis of SRCR-like domains

The peptide sequences of the SRCR-like domains from *AQ*, *SC*, and *XT* were queried against each other using Blastp [62]. A threshold of ≥ 90 % positive-scoring matches between two domains was used to identify homology. The Markov Cluster (MCL) Algorithm [68] was used to cluster the SRCR-like domains, with the Blastp bit score applied as a similarity metric. The SRCR-like domain sequences from each cluster were aligned using MAFFT v7.123b [69], with the extension penalty parameter and maximum iterations set to 0.123 and 3, respectively. We computed the amino acid frequency at each aligned position using a custom Python script.

### Microbial community analysis

For Illumina reads, the low-quality ends (from the first base with Q-score < 20, which correspond to an error probability of 0.01, to the 3’ end) and sequencing adapters were trimmed using custom Java scripts.

The preprocessed metatranscriptome reads were further processed to remove any rRNA fragments, using riboPicker v0.4.3 [70] with thresholds of 90 % alignment coverage and 90 % alignment identity. Blastx [62] was then used to align the reads against the nr database to obtain the best-hit sequence for each aligned read. To create each reference sequence, we measured the similarities between extracted sequences using Blastp, and clustered them into homology groups using MCL [68] with the inflation parameter set to 3.6 and the other parameters set at their default values.

To quantify the expression level of each homology group per sample per sponge, we summed the numbers of reads whose best hits were assigned to each homology group, then further quantile-normalised the read counts across samples using the preprocessCore package in R [71]. The differentially expressed homology groups between two sponges were obtained using GFOLD v1.1.2 [72], with an expected false discovery rate (FDR) ≤ 0.05.

Representative sequences of each homology group were annotated with respect to PFAM [73] domains using InterProScan v5. RC7 [66]. The GO terms for each domain were also obtained [74]. The statistical significance of each domain and the GO term enrichments observed among the differentially expressed homology groups were assessed based on the cumulative hypergeometric distributions and FDRs (≤0.05), which were calculated with a custom R script. Fourteen and 20 PFAM domains were found to be statistically significant in the *SC* and *XT* metatranscriptome datasets, respectively (Fig. 4). SUPERFAMILY [13] domains were annotated in the same way (Additional file 14).

### Availability of supporting data

The generated sequencing datasets for *SC* and *XT* are publicly available under NCBI BioProject IDs PRJNA254402 and PRJNA254412, respectively. The genome assemblies, transcripts, and coding sequences for both sponges are available at http://sc.reefgenomics.org and http://xt.reefgenomics.org.

## Additional files

Additional file 1: Transmission electron microscope (TEM) images of studied sponges. (a–d) TEM images of *Stylissa carteri* (*SC*). A number of the *SC* cells are packed with vesicle-like inclusions. Microbes are not observed in the mesohyl. Spongin (spincy lines), which gives structure to the sponge tissues, and choanocytes are observed. (e–j) TEM images of *Xestospongia testudinaria* (*XT*). Archaeocytes are seen to be engulfing bacteria for digestion. Unique sponge symbionts, such as cyanobacteria with thylakoid membranes, are frequently observed. Spirochaetes are also observed (arrow in j). Abbreviations: ECM, extracellular matrix; HSC, host sponge cell; ST, storage cell; n, nucleus; b, bacterium; ub, undigested bacterium; db, digested bacterium; and f, flagella. (PDF 7354 kb) Additional file 2: Sources and statistics of sequences used for the hologenome analysis. (XLSX 19 kb) Additional file 3: Detailed statistics of our sponge genomes and transcriptomes. (PDF 263 kb) Additional file 4: Comparison of gene annotation quality among sponge dataset. Transcript models of 16 sponge species are compared to address the quality of gene annotation. (PDF 60 kb) Additional file 5: Over- or under-represented Superfamily domains in *SC* and *XT*. Superfamily domains that are unusually enriched in *SC* and *XT*. Domains with |deviation| > 0.5 are shown. (PDF 64 kb) Additional file 6: Superfamily domains related to innate immunity and symbiosis. Sixteen species were compared; their NCBI taxonomic IDs are given in brackets. (XLSX 72 kb) Additional file 7: Supplementary information describing relationship between *Xestospongia testudinaria* and *Xestospongia muta*, antimicrobial peptides, and evolutionary rates of innate immune domains in analyzed sponge genomes. (PDF 790 kb) Additional file 8: Statistics of compiled AMPs for broad taxonomic group. (PDF 34 kb) Additional file 9: Taxonomic origins of the AMPs that were successfully aligned to the sponge genomes. (PDF 47 kb) Additional file 10: Antimicrobial peptides encoded in the sponge genomes. (PDF 78 kb) Additional file 11: The number of AMPs with different biological activities on the sponge genomes. (PDF 56 kb) Additional file 12: Evolutionary rates of selected domains. (a) Boxplot representing the distribution of the mean Ka/Ks for each protein domain between sponge pairs. (b) The mean Ka/Ks of each protein domain is shown. This analysis was restricted to the protein domains given in Fig. 2 that passed our quality control step (see Methods). (PDF 194 kb) Additional file 13: Expression levels of protein domains in the metatranscriptome dataset. PFAM and SUPERFAMILY domains are ranked by their expression levels, which were quantile-normalised and then summed. (XLSX 462 kb) Additional file 14: SUPERFAMILY domains enriched among the genes found to be differentially expressed in each sponge metatranscriptome. (PDF 50 kb) Additional file 15: Properties of gene models. Gene models were tagged based on the presence of eukaryotic or prokaryotic sequences, as assessed by comparison to the NCBI nr database (see Methods). The properties of the gene models were analysed based on the numbers of exons and the transcriptional expression levels. (PDF 481 kb)

## Abbreviations

AED: Annotation edit distance
AQ: *Amphimedon queenslandica*
ASW: Artificial seawater
CEGs: Core eukaryotic genes
CMF: Calcium-magnesium-free
DAMPs: Endogenous danger-associated molecular patterns
GO: Gene ontology
HMA: High microbial abundance
HMG: High-mobility group
LDL: Low-density lipoprotein
LMA: Low microbial abundance
MAMPs: Microbe-associated molecular patterns
NLR: Leucine-rich repeat
PAMPs: Pathogen-associated molecular patterns
SC: *Stylissa carteri*
SRCR: Scavenger receptor cysteine-rich
XT: *Xestospongia testudinaria*
