# Supplementary figures and images for: Hologenome analysis of two marine sponges with different microbiomes
Source: BMC Genomics. 2016 Feb 29;17:158. doi: 10.1186/s12864-016-2501-0 (PMC4772301; doi:10.1186/s12864-016-2501-0)

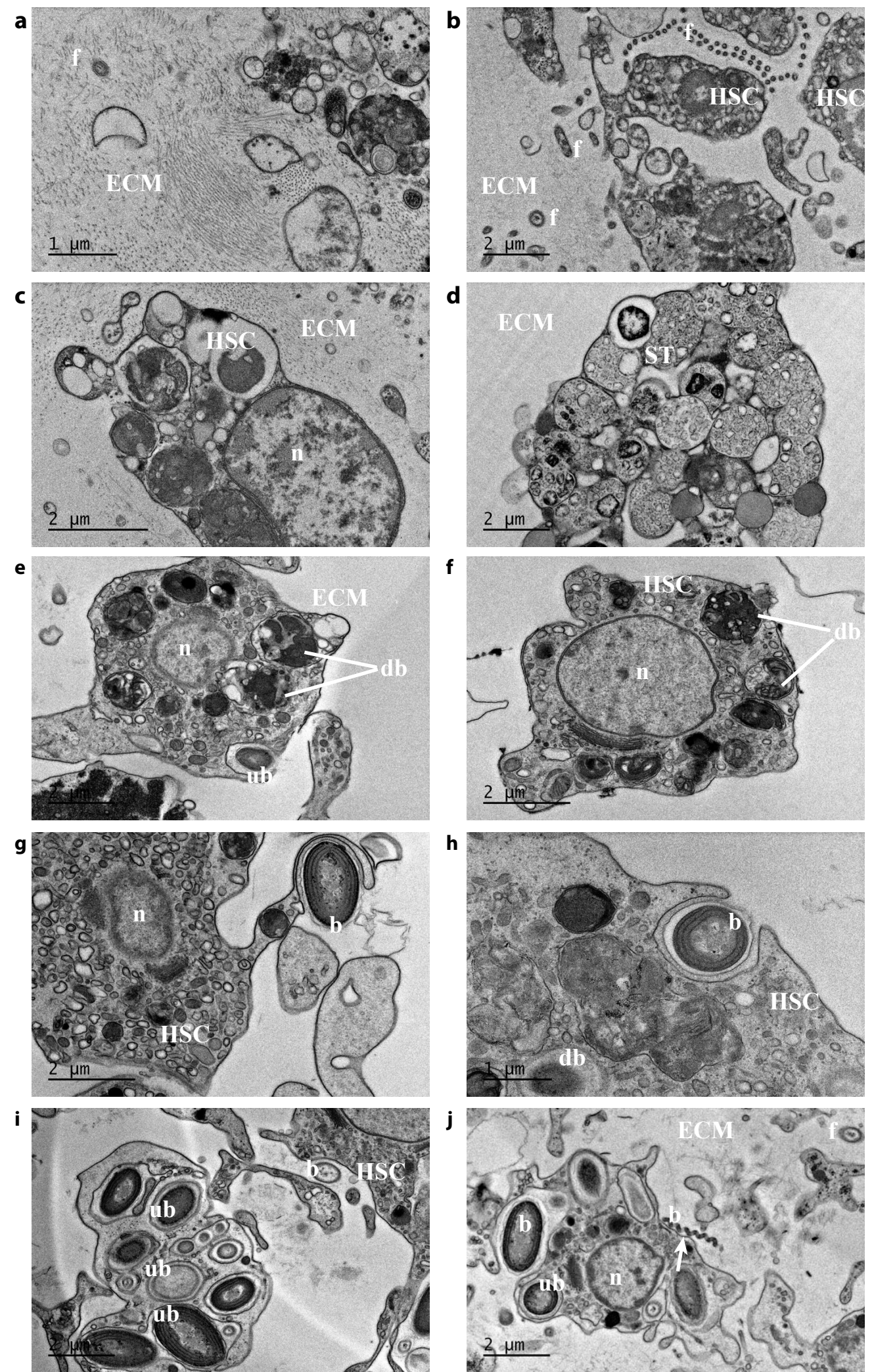

Supplement: Additional file 1: — Transmission electron microscope (TEM) images of studied sponges. (a–d) TEM images of Stylissa carteri (SC). A number of the SC cells are packed with vesicle-like inclusions. Microbes are not observed in the mesohyl. Spongin (spincy lines), which gives structure to the sponge tissues, and choanocytes are observed. (e–j) TEM images of Xestospongia testudinaria (XT). Archaeocytes are seen to be engulfing bacteria for digestion. Unique sponge symbionts, such as cyanobacteria with thylakoid membranes, are frequently observed. Spirochaetes are also observed (arrow in j). Abbreviations: ECM, extracellular matrix; HSC, host sponge cell; ST, storage cell; n, nucleus; b, bacterium; ub, undigested bacterium; db, digested bacterium; and f, flagella. (PDF 7354 kb) [file 12864_2016_2501_MOESM1_ESM.pdf]

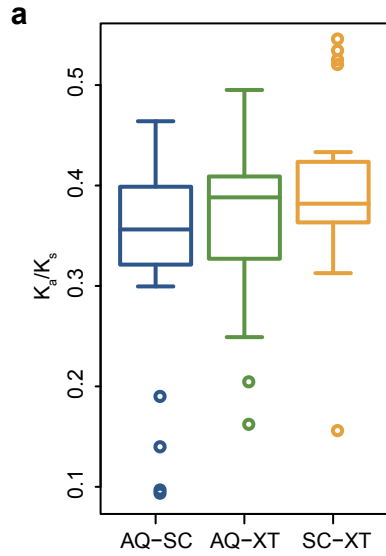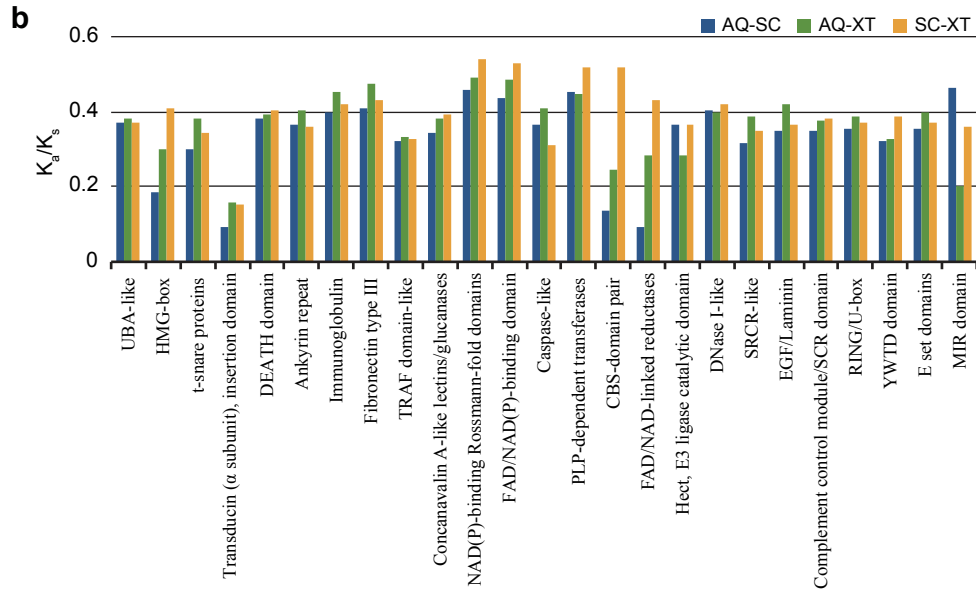

Supplement: Additional file 12: — Evolutionary rates of selected domains. (a) Boxplot representing the distribution of the mean Ka/Ks for each protein domain between sponge pairs. (b) The mean Ka/Ks of each protein domain is shown. This analysis was restricted to the protein domains given in Fig. 2 that passed our quality control step (see Methods). (PDF 194 kb) [file 12864_2016_2501_MOESM12_ESM.pdf]

Eukaryotes Prokaryotes No hit

### Gene tag

SC

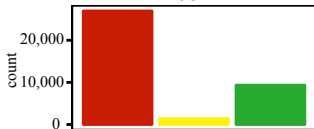

XT

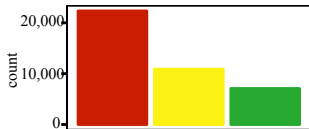

### Gene exon count

SC

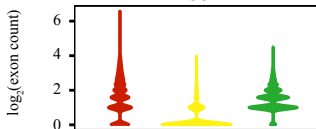

XT

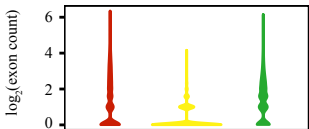

### Gene expression level

SC

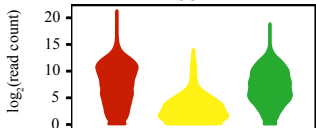

XT

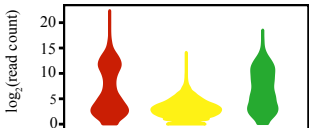

Supplement: Additional file 15: — Properties of gene models. Gene models were tagged based on the presence of eukaryotic or prokaryotic sequences, as assessed by comparison to the NCBI nr database (see Methods). The properties of the gene models were analysed based on the numbers of exons and the transcriptional expression levels. (PDF 481 kb) [file 12864_2016_2501_MOESM15_ESM.pdf]
