# Supplementary material for: Hologenome analysis of two marine sponges with different microbiomes
Source: BMC Genomics. 2016 Feb 29;17:158. doi: 10.1186/s12864-016-2501-0 (PMC4772301; doi:10.1186/s12864-016-2501-0)
Supplement: Additional file 3: — Detailed statistics of our sponge genomes and transcriptomes. (PDF 263 kb) [file 12864_2016_2501_MOESM3_ESM.pdf]

de novo genome assembly (raw assembly from Velvet)

	number of scaffolds	min. length (bp)	N50 (bp)	N20 (bp)	max. length (bp)	sum (Mbp)
SC	358,309	200	4,522	59,233	965,826	508.82
XT	816,724	200	852	6,379	1,572,474	492.59

de novo genome assembly (final version)

	number of scaffolds	min. length (bp)	N50 (bp)	N20 (bp)	max. length (bp)	sum (Mbp)
SC	97,497	800	10,236	83,618	965,826	418.92
XT	97,640	800	4,078	29,235	1,572,474	257.96

de novo transcriptome assembly

	number of contigs	min. length (bp)	N50 (bp)	N20 (bp)	max. length (bp)	sum (Mbp)
SC	326,400	200	444	800	48,514	134.38
XT	346,768	200	491	958	48,514	151.8

annotated transcripts from the gene models

	number of contigs	min. length (bp)	N50 (bp)	N20 (bp)	max. length (bp)	sum (Mbp)
SC (eukaryotic)	26,967	63	1,176	2,184	18,322	25.29
SC (prokaryotic)	1,385	141	1,236	2,040	6,708	1.45
SC (unknown)	9,343	61	714	1,278	6,915	5.67
XT (eukaryotic)	22,337	93	1,312	2,427	21,282	24.09
XT (prokaryotic)	10,879	117	1,200	2,055	8,798	11.24
XT (unknown)	7,082	45	1,043	1,908	11,070	5.85
