# Supplementary material for: Hologenome analysis of two marine sponges with different microbiomes
Source: BMC Genomics. 2016 Feb 29;17:158. doi: 10.1186/s12864-016-2501-0 (PMC4772301; doi:10.1186/s12864-016-2501-0)
Supplement: Additional file 4: — Comparison of gene annotation quality among sponge dataset. Transcript models of 16 sponge species are compared to address the quality of gene annotation. (PDF 60 kb) [file 12864_2016_2501_MOESM4_ESM.pdf]

Species name	total # of CDSs	# of CEGMA core genes (% among 458 CEGMA core genes)	CDSs with ≥1 protein domains[a]	total # of protein domain[b]	# of unique protein domain[c]
Amphimedon queenslandica	30,060	451 (98.47)	17,567	32,326	1,032
Aphrocallistes vastus	17,512	448 (97.82)	9,146	15,966	920
Chondrilla nucula	28,667	440 (96.07)	12,145	16,841	980
Corticium candelabrum	53,195	440 (96.07)	21,617	29,564	1,134
Ephydatia muelleri	34,571	441 (96.29)	14,075	24,857	968
Ircinia fasciculata	24,780	435 (94.98)	10,836	13,515	937
Leucosolenia complicata	73,768	449 (98.03)	35,397	52,633	1,076
Oscarella carmela	30,420	454 (99.13)	14,603	27,515	1,010
Oscarella sp	28,366	452 (98.69)	12,443	17,455	959
Petrosia ficiformis	30,228	435 (94.98)	12,476	17,704	939
Pseudospongosorites suberitoides	16,802	428 (93.45)	6,956	8,420	854
Spongilla lacustris	21,957	429 (93.67)	9,638	12,980	897
Stylissa carteri	26,967	435 (94.98)	17,074	28,027	1,007
Sycon ciliatum	33,929	446 (97.38)	14,763	30,709	983
Sycon coactum	37,923	444 (96.94)	14,319	22,635	974
Xestospongia testudinaria	22,337	432 (94.32)	17,664	29,156	1,119

[a,b,c] SUPERFAMILY domains were used to calculate these numbers.
