# Supplementary material for: Hologenome analysis of two marine sponges with different microbiomes
Source: BMC Genomics. 2016 Feb 29;17:158. doi: 10.1186/s12864-016-2501-0 (PMC4772301; doi:10.1186/s12864-016-2501-0)
Supplement: Additional file 5: — Over- or under-represented Superfamily domains in SC and XT. Superfamily domains that are unusually enriched in SC and XT. Domains with |deviation| > 0.5 are shown. (PDF 64 kb) [file 12864_2016_2501_MOESM5_ESM.pdf]

species	Deviation	Log Ratio	Domains	Average	Superfamily
SC	7.72%	4.12	2114	27.5	SRCR-like
	3.79%	1.71	1246	160.8	Ankyrin repeat
	2.47%	0.68	1344	503	Protein kinase-like (PK-like)
	2.30%	3.04	650	26.7	TRAF domain-like
	1.95%	1.38	701	145.9	Fibronectin type III
	1.89%	1.25	710	170.4	EGF/Laminin
	1.80%	3.23	503	13.5	CalX-like
	1.57%	0.68	852	367.8	Immunoglobulin
	1.47%	1.69	482	74.6	Complement control module/SCR domain
	1.37%	2.68	394	23.3	DEATH domain
	0.83%	0.15	1641	911.6	P-loop containing nucleoside triphosphate hydrolases
	0.78%	0.52	516	233.5	RING/U-box
	0.72%	1.5	246	40.9	Kelch motif
	0.66%	0.87	303	92.3	E set domains
	0.59%	2.44	172	10.6	NHL repeat
	0.52%	0.6	305	147.3	Ribonuclease H-like
	-0.57%	-1.23	60	175.2	Family A G protein-coupled receptor-like
	-0.58%	-0.71	148	191	alpha/beta-Hydrolases
	-0.64%	-0.7	166	231.4	RNA-binding domain, RBD
	-0.91%	-0.77	208	288.9	ARM repeat
	-2.45%	-1.93	109	625	beta-beta-alpha zinc fingers
XT	3.52%	0.51	1959	911.6	P-loop containing nucleoside triphosphate hydrolases
	3.50%	3.35	808	27.5	SRCR-like
	3.10%	1.05	1062	266.1	NAD(P)-binding Rossmann-fold domains
	1.79%	1.14	586	160.8	Ankyrin repeat
	1.30%	1.44	377	64.2	PLP-dependent transferases
	1.08%	2.02	275	27.8	ClpP/crotonase
	0.95%	2.13	238	21.1	Alkaline phosphatase-like
	0.79%	0.79	319	103.3	Nucleic acid-binding proteins
	0.72%	1.58	200	29.1	Glutathione synthetase ATP-binding domain-like
	0.69%	1.54	194	28.1	Thiamin diphosphate-binding fold (THDP-binding)
	0.59%	1.25	184	34.7	Nucleotidylyl transferase
	0.56%	0.55	294	117.7	FAD/NAD(P)-binding domain
	0.55%	1.86	143	23.3	DEATH domain
	0.52%	0.59	262	145.9	Fibronectin type III
	0.50%	1.2	156	29.8	Class II aaRS and biotin synthetases
	0.50%	1	175	44	Ribosomal protein S5 domain 2-like
	-0.61%	-0.66	143	231.4	RNA-binding domain, RBD

-0.64%	-1.65	32	175.2	Family A G protein-coupled receptor-like
-0.84%	-0.68	189	288.9	ARM repeat
-0.88%	-0.99	114	212.6	MFS general substrate transporter
-0.90%	-0.44	360	503	Protein kinase-like (PK-like)
-2.52%	-2.14	73	625	beta-beta-alpha zinc fingers
