# Supplementary material for: Hologenome analysis of two marine sponges with different microbiomes
Source: BMC Genomics. 2016 Feb 29;17:158. doi: 10.1186/s12864-016-2501-0 (PMC4772301; doi:10.1186/s12864-016-2501-0)
Supplement: Additional file 7: — Supplementary information describing relationship between Xestospongia testudinaria and Xestospongia muta, antimicrobial peptides, and evolutionary rates of innate immune domains in analyzed sponge genomes. (PDF 790 kb) [file 12864_2016_2501_MOESM7_ESM.pdf]

Supplementary Information

Similarity of genome sizes between *Xestospongia testudinaria* and *Xestospongia muta*

The flow-cytometry-determined genome size of 0.165 pg for *XT* was similar to the Feulgen-image-analysis-densitometry (FIAD)-determined genome size of 0.14 pg previously reported for *Xestospongia muta* (*XM*) [1]. Since flow-cytometry-based estimates of genome sizes are an average of ~ 18 % higher than FIAD-based estimates [1], the flow-cytometry-estimated genome size of *XM* would be roughly 0.165 pg, which is the same as that of *XT*. These two ubiquitous sponges are found in different seas (*XM* in the Atlantic Ocean and *XT* in the Indo-Pacific Ocean and Red Sea), but they are similar in their morphologies and microbial communities [2, 3]. Thus, we speculate these closely related species might have diverged due to the imposition of geographic barriers (allopatric speciation) when the Isthmus of Panama separated the two oceans about 3 million years ago [4].

Identification of antimicrobial peptides (AMPs) in sponges

Since host-generated AMPs can prevent colonisation by harmful microbes and exert species-specific control on microbial communities [5], we investigated AMPs in the studied sponges.

We integrated three comprehensive AMP databases, AMPer [6], APD2 [7], and DAMPD [8], and selected only the experimentally validated AMPs to increase the reliability of their prediction in sponges. Only one AMP was kept when 100 % identical AMPs exist.

This resulted in 2,431, 963, and 779 AMPs from 2,436, 1,045, and 792 AMPs cataloged in the APD2, CMDR, and DAMPD databases, respectively.

Due to the presence of ambiguous phylogenetic information in the original databases, AMPs were classified into 10 broad taxonomic classes (Amphibians, Birds, Fish, Fungi, Invertebrates, Mammals, Plants, Prokaryotes, Reptiles, and Others) according to their taxonomy labels in the FASTA headers. AMPs that lacked taxonomic information and were found with low frequency (< 5 times) were labeled as "Others" (Additional data file 8).

We used tBlastn to identify AMPs in the genomes of *AQ*, *SC*, and *XT*. An e-value threshold of 10^{-6} was used as a similarity cut-off, and all other parameters were used at their default values. The AMPs predicted in the three sponge genomes are shown in Additional data file 9 and 10.

We identified a large proportion of invertebrate AMPs in all three sponges (51 % of the total hits); this was a considerably higher proportion than that found in the merged database (13.9 %), presumably reflecting the phylogenetic similarity between sponges and other invertebrates. *SC* and *XT* harbored the most and least AMPs, respectively, suggesting that LMA sponges have a better microbe-clearing mechanism related to these peptides, compared to HMA sponges.

The APD2 database has several useful attributes, including data on the activity of each AMP. Our analysis of these AMP characteristics revealed that the sponge genomes had abundant antibacterial and antifungal peptides (Additional data file 11). This finding highlights the possibility that sponge AMPs could be used in commercial products, such as therapeutic or antifouling compounds.

Evolutionary rate of protein domains

We calculated the synonymous to non-synonymous mutation rate (K_a/K_s) for each protein domain shown in Figure 2. Genes found to contain each SUPERFAMILY domain were selected for *AQ*, *SC*, and *XT*. All gene pairs from different sponges were subjected to pairwise alignment using transAlign [9]. Poorly aligned gene pairs (K_a or $K_s > 5$) were excluded. K_a/K_s values were calculated using SeqInR [10], and the mean K_a/K_s value per species pair per protein domain was obtained (Additional data file 12). Our results indicated that the LMA sponges shared similar evolutionary patterns, which were significantly different from that of the HMA sponge (P=0.028 by Mann-Whitney test). This further strengthens our contention that microbial abundance is related to the evolution of sponge genomes.

References

1. Jeffery NW, Jardine CB, Gregory TR: **A first exploration of genome size diversity in sponges**. *Genome* 2013, **56**(8):451-456.
2. Montalvo NF, Hill RT: **Sponge-associated bacteria are strictly maintained in two closely related but geographically distant sponge hosts**. *Appl Environ Microbiol* 2011, **77**(20):7207-7216.
3. Schmitt S, Tsai P, Bell J, Fromont J, Ilan M, Lindquist N, Perez T, Rodrigo A, Schupp PJ, Vacelet J *et al*: **Assessing the complex sponge microbiota: core, variable and species-specific bacterial communities in marine sponges**. *Isme J* 2012, **6**(3):564-576.
4. Keigwin L: **Isotopic paleoceanography of the Caribbean and East pacific: role of panama uplift in late neogene time**. *Science* 1982, **217**(4557):350-353.
5. Franzenburg S, Walter J, Kunzel S, Wang J, Baines JF, Bosch TC, Fraune S: **Distinct antimicrobial peptide expression determines host species-specific bacterial associations**. *Proc Natl Acad Sci U S A* 2013, **110**(39):E3730-3738.
6. Fjell CD, Hancock RE, Cherkasov A: **AMPer: a database and an automated discovery tool for antimicrobial peptides**. *Bioinformatics* 2007, **23**(9):1148-1155.
7. Wang G, Li X, Wang Z: **APD2: the updated antimicrobial peptide database and its application in peptide design**. *Nucleic Acids Res* 2009, **37**(Database issue):D933-937.

8. Seshadri Sundararajan V, Gabere MN, Pretorius A, Adam S, Christoffels A, Lehvaslaiho M, Archer JA, Bajic VB: **DAMPD: a manually curated antimicrobial peptide database**. *Nucleic Acids Res* 2012, **40**(Database issue):D1108-1112.
9. Bininda-Emonds OR: **transAlign: using amino acids to facilitate the multiple alignment of protein-coding DNA sequences**. *BMC bioinformatics* 2005, **6**:156.
10. Charif D, Lobry JR: **SeqinR 1.0-2: a contributed package to the R project for statistical computing devoted to biological sequences retrieval and analysis**. *Structural Approaches to Sequence Evolution* 2007, **1**:207-232.
