# Supplementary material for: Hologenome analysis of two marine sponges with different microbiomes
Source: BMC Genomics. 2016 Feb 29;17:158. doi: 10.1186/s12864-016-2501-0 (PMC4772301; doi:10.1186/s12864-016-2501-0)
Supplement: Additional file 8: — Statistics of compiled AMPs for broad taxonomic group. (PDF 34 kb) [file 12864_2016_2501_MOESM8_ESM.pdf]

Taxonomy	# of AMPs	Percentage	Mean Length (aas)	Minimum Length (aas)	Maximum Length (aas)
Amphibians	1,310	31.39	29.56	7	303
Birds	54	1.29	55.94	8	251
Fish	87	2.08	28.55	12	88
Fungi	30	0.72	45.07	10	97
Invertebrates	518	12.41	43.64	8	394
Mammals	668	16.01	55.59	5	710
Others	799	19.15	57.18	6	625
Plants	418	10.02	46.4	7	708
Prokaryotes	277	6.64	39.58	2	339
Reptiles	12	0.29	36	22	50
Total	4,173	100	43.56	2	710
