# Supplementary material for: Hologenome analysis of two marine sponges with different microbiomes
Source: BMC Genomics. 2016 Feb 29;17:158. doi: 10.1186/s12864-016-2501-0 (PMC4772301; doi:10.1186/s12864-016-2501-0)
Supplement: Additional file 9: — Taxonomic origins of the AMPs that were successfully aligned to the sponge genomes. (PDF 47 kb) [file 12864_2016_2501_MOESM9_ESM.pdf]

Taxonmy	*AQ*	*SC*	*XT*	Total Number of Hits
Amphibians	0	0	0	0
Birds	11	13	1	25
Fish	3	7	1	11
Fungi	0	0	0	0
Invertebrates	122	286	52	460
Mammals	103	60	57	220
Others	35	56	91	182
Plants	1	1	1	3
Prokaryotes	0	2	0	2
Reptiles	0	0	0	0
Total Number of Hits	275	425	203	903
