# Supplementary material for: Hologenome analysis of two marine sponges with different microbiomes
Source: BMC Genomics. 2016 Feb 29;17:158. doi: 10.1186/s12864-016-2501-0 (PMC4772301; doi:10.1186/s12864-016-2501-0)
Supplement: Additional file 10: — Antimicrobial peptides encoded in the sponge genomes. (PDF 78 kb) [file 12864_2016_2501_MOESM10_ESM.pdf]

species	database reference ID	description	group Label	# of hits
AQ	APD2030	cgUbiquitin	Invertebrates	67
	APD2388	Bovine Pancreatic Trypsin Inhibitor	Mammals	31
	APD307	Buforin I	Mammals	3
	APD489	Hipposin	Fish	3
	CMDR126	Lipopolysaccharide-binding protein	Mammals	1
	CMDR127	lipopolysaccharide binding protein	Mammals	1
	CMDR128	Bactericidal permeability-increasing protein	Mammals	1
	CMDR129	bactericidal permeability increasing protein precursor	Mammals	1
	CMDR130	Bactericidal permeability-increasing protein	Mammals	2
	CMDR131	lipopolysaccharide-binding protein precursor	Mammals	1
	CMDR132	Chain A of Lipopolysaccharide Binding Protein	Mammals	1
	CMDR133	Lipopolysaccharide-binding protein	Mammals	1
	CMDR153	Hipposin	Others	3
	CMDR570	Hemolin	Others	4
	CMDR571	Hemolin	Invertebrates	6
	CMDR599	Buforin-1	Others	3
	DAMPD112	H2A_BUFBG	Others	3
	DAMPD116	Histone H2B type F-S	Mammals	11
	DAMPD117	H2A_HIPHI	Others	3
	DAMPD118	H2B1C_HUMAN	Mammals	11
	DAMPD154	Lectin L6	Invertebrates	30
	DAMPD183	H2B_LITVA	Invertebrates	11
	DAMPD184	H4_LITVA	Invertebrates	2
	DAMPD20	H2B1K_HUMAN	Mammals	11
	DAMPD236	H2B2E_HUMAN	Mammals	11
	DAMPD247	Lysozyme	Others	2
	DAMPD256	Bactericidal permeability-increasing protein	Mammals	1
	DAMPD261	H2A_LITVA	Invertebrates	6
	DAMPD264	H2B_RHAS	Others	11
	DAMPD27	H2A_ONCMY	Others	6
	DAMPD32	Pathogenesis-related leaf protein 6	Plants	1
	DAMPD38	H2B1J_HUMAN	Mammals	11
	DAMPD45	H2B1_CHICK	Birds	11
	DAMPD73	Acyl-CoA-binding protein	Mammals	3
	DAMPD86	Bactericidal permeability-increasing protein	Mammals	1
SC	APD1575	Thrombin-derived C-terminal Peptide	Mammals	1
	APD2030	cgUbiquitin	Invertebrates	248
	APD2131	Cyanovirin-N	Prokaryotes	2
	APD2388	Bovine Pancreatic Trypsin Inhibitor	Mammals	21
	APD307	Buforin I	Mammals	7

	APD489	Hipposin	Fish	7
	CMDR153	Hipposin	Others	7
	CMDR599	Buforin-1	Others	7
	DAMPD112	H2A_BUFBG	Others	7
	DAMPD113	RL1_HELPY	Others	2
	DAMPD116	H2BFS_HUMAN	Mammals	6
	DAMPD117	H2A_HIPHI	Others	7
	DAMPD118	H2B1C_HUMAN	Mammals	6
	DAMPD154	LEC6_TACTR	Invertebrates	18
	DAMPD183	H2B_LITVA	Invertebrates	6
	DAMPD184	H4_LITVA	Invertebrates	4
	DAMPD20	H2B1K_HUMAN	Mammals	6
	DAMPD236	H2B2E_HUMAN	Mammals	6
	DAMPD253	L-amino-acid oxidase (Fragments)	Others	2
	DAMPD254	L-amino-acid oxidase	Others	8
	DAMPD261	H2A_LITVA	Invertebrates	10
	DAMPD264	H2B_RHASC	Others	6
	DAMPD27	H2A_ONCMY	Others	10
	DAMPD32	Pathogenesis-related leaf protein 6	Plants	1
	DAMPD38	H2B1J_HUMAN	Mammals	6
	DAMPD45	H2B1_CHICK	Birds	6
	DAMPD461	Cathelicidin-B1	Birds	7
	DAMPD73	ACBP_PIG	Mammals	1
XT	APD1575	Thrombin-derived C-terminal Peptide	Mammals	3
	APD2030	cgUbiquitin	Invertebrates	28
	APD2096	Ubiquicidin	Mammals	1
	APD2388	Bovine Pancreatic Trypsin Inhibitor	Mammals	39
	APD307	Buforin I	Mammals	1
	APD489	Hipposin	Fish	1
	CMDR129	bactericidal permeability increasing protein precursor	Mammals	2
	CMDR130	Bactericidal permeability-increasing protein	Mammals	2
	CMDR131	lipopolysaccharide-binding protein precursor	Mammals	1
	CMDR153	Hipposin	Others	1
	CMDR570	Hemolin	Others	1
	CMDR571	Hemolin	Invertebrates	4
	CMDR599	Buforin-1	Others	1
	DAMPD112	H2A_BUFBG	Others	1
	DAMPD113	RL1_HELPY	Others	58
	DAMPD116	H2BFS_HUMAN	Mammals	1
	DAMPD117	H2A_HIPHI	Others	1
	DAMPD118	H2B1C_HUMAN	Mammals	1
	DAMPD154	LEC6_TACTR	Invertebrates	13
	DAMPD183	H2B_LITVA	Invertebrates	1

DAMPD184	H4_LITVA	Invertebrates	3
DAMPD20	H2B1K_HUMAN	Mammals	1
DAMPD236	H2B2E_HUMAN	Mammals	1
DAMPD253	L-amino-acid oxidase (Fragments)	Others	8
DAMPD254	L-amino-acid oxidase	Others	16
DAMPD261	H2A_LITVA	Invertebrates	3
DAMPD264	H2B_RHASC	Others	1
DAMPD27	H2A_ONCMY	Others	3
DAMPD32	Pathogenesis-related leaf protein 6	Plants	1
DAMPD38	H2B1J_HUMAN	Mammals	1
DAMPD45	H2B1_CHICK	Birds	1
DAMPD86	Bactericidal permeability-increasing protein	Mammals	2
DAMPD89	Azurocidin	Mammals	1
