# Supplementary material for: Hologenome analysis of two marine sponges with different microbiomes
Source: BMC Genomics. 2016 Feb 29;17:158. doi: 10.1186/s12864-016-2501-0 (PMC4772301; doi:10.1186/s12864-016-2501-0)
Supplement: Additional file 11: — The number of AMPs with different biological activities on the sponge genomes. (PDF 56 kb) [file 12864_2016_2501_MOESM11_ESM.pdf]

activity	count in APD2	% in APD2	the number of hits on sponge genomes		
			AQ	*SC*	*XT*
Antibacterial Peptides	2,003	82.39	73	263	34
Antiviral Peptides	147	6.05	0	2	0
Antifungal Peptides	896	36.86	34	28	40
Antiparasitic Peptides	62	2.55	0	0	0
Anticancer/tumor Peptides	170	6.99	0	0	0
Anti-protist Peptides	4	0.16	0	0	0
Insecticidal Peptides	23	0.95	0	0	0
Spermicidal Peptides	10	0.41	0	0	0
Anti-HIV-1 Peptides	93	3.83	0	2	0
Antioxidant Peptides	12	0.49	0	0	0
Enzyme/protease inhibitory peptides	9	0.37	0	0	0
Chemotactic Peptides	50	2.06	0	0	0
total number of hits			107	295	74
