# Supplementary material for: Hologenome analysis of two marine sponges with different microbiomes
Source: BMC Genomics. 2016 Feb 29;17:158. doi: 10.1186/s12864-016-2501-0 (PMC4772301; doi:10.1186/s12864-016-2501-0)
Supplement: Additional file 14: — SUPERFAMILY domains enriched among the genes found to be differentially expressed in each sponge metatranscriptome. (PDF 50 kb) [file 12864_2016_2501_MOESM14_ESM.pdf]

species	domain ID	description	adjusted p-value
SC	SSF57756	Retrovirus zinc finger-like domains	1.82E-04
	SSF81483	Bacterial photosystem II reaction centre, L and M subunits	2.28E-04
	SSF56672	DNA/RNA polymerases	2.28E-04
	SSF47836	Retroviral matrix proteins	6.67E-03
	SSF47943	Retrovirus capsid protein, N-terminal core domain	1.94E-02
	SSF103501	Respiratory nitrate reductase 1 gamma chain	1.94E-02
	SSF47240	Ferritin-like	2.40E-02
	SSF161077	Photosystem II antenna protein-like	4.01E-02
	SSF47353	Retrovirus capsid dimerization domain-like	4.01E-02
	SSF48173	Cryptochrome/photolyase FAD-binding domain	4.01E-02
	SSF81558	Photosystem I subunits PsaA/PsaB	4.01E-02
XT	SSF51658	Xylose isomerase-like	1.12E-03
